# Role of PGC‐1α in fiber type conversion in the palatopharyngeus muscle of OSA patients

**DOI:** 10.1002/jcla.24551

**Published:** 2022-06-12

**Authors:** Ling Chen, Yongqing Shen, Hao Xiong, Zhong Guan, Yu Si, Haifeng Liang, Wenying Zhu, Qian Cai

**Affiliations:** ^1^ Department of Otolaryngology‐Head and Neck, Sun Yat‐sen Memorial Hospital Sun Yat‐sen University Guang zhou China

**Keywords:** intermittent hypoxia, OSA, PGC‐1α, skeletal muscle fiber type

## Abstract

**Background:**

Obstructive sleep apnea (OSA) has a high incidence and is harmful to health. It is characterized by repeated collapse of the upper airway. However, the mechanism underlying upper airway collapse is unclear.

**Methods:**

Patients with OSA and chronic tonsillitis were studied. Pathological changes in palatopharyngeus muscle were detected. The expression of peroxisome proliferator‐activated receptor‐γ co‐activator‐1α (PGC‐1α) and nuclear respiratory factor‐1 (NRF‐1) in muscles was detected by PCR and Western blotting. Immunofluorescence staining was used to detect the expression of type I and type II myofibril.

**Results:**

The structure of the palatopharyngeus muscle was changed, and the expression of PGC‐1α and NRF‐1 was decreased in the OSA group compared with that in the control group. The expression of PGC‐1α, NRF‐1, and type I myofibril in C2C12 myoblasts was decreased by intermittent hypoxia exposure. The expression of type I myofibril was decreased when knocking down PGC‐1α.

**Conclusion:**

OSA patients exhibited pathological damage in palatopharyngeus muscle. PGC‐1α was involved in the fiber type conversion in palatopharyngeus muscle caused by intermittent hypoxia.

## Graphical abstract

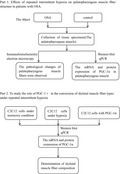

## 1 INTRODUCTION

Obstructive sleep apnea (OSA) has a high incidence of 2%–4% in the population.^1^ Chronic intermittent hypoxia (CIH) is the main pathophysiological characteristic of OSA. Current studies have shown that OSA is caused by a combination of many pathological factors, and it is characterized by hypoxia and a series of secondary problems caused by the repeated collapse of the upper airway.^2^ The mechanism underlying upper airway collapse is still unclear. Studying this mechanism is helpful for identifying key targets for controlling and treating upper airway collapse, which is of great significance for the prevention and treatment of OSA.

Skeletal muscle is composed of different muscle fiber types in a specific proportion. Muscle fibers can be divided into type I fibers and type II fibers according to the type of myosin heavy chain (MHC). Type I fibers, also called “slow oxidized fibers,” contain myosin of slow muscle fibers (Myhc slow) and exhibit slow contraction speeds and strong fatigue resistance. Type II fibers are called “fast glycolytic fibers,” and they contain Myhc fast and mainly obtain energy from anaerobic glycolysis. The tension and contraction of muscle fibers play important roles in maintaining the upper airway in an open position.^3^

Previous studies have shown that in OSA, palatopharyngeus muscle exhibit pathological injuries, including muscle fibers atrophy, structural disorder, and type I fibers reduction,^4^, ^5^ which lead to changes in function of the palatopharyngeus muscle, collapse of the upper airway and exacerbation of hypoxia.

Peroxisome proliferator‐activated receptor‐γ (PPAR‐γ) is a ligand‐activated nuclear transcription factor that belongs to the nuclear hormone receptor superfamily. Peroxisome proliferator‐activated receptor‐γ co‐activator‐1α (PGC‐1α) is a coactivator of PPAR‐γ, which participates in the transcriptional regulation of PPAR‐γ. It is a key factor in mitochondrial biosynthesis and highly expressed in skeletal muscle, especially in oxidized muscle fibers. Lin et al. showed that PGC‐1α promoted skeletal muscle fiber conversion.^6^ Studies have shown that hypoxia can down‐regulate the expression of PPAR‐γ in mouse pulmonary artery smooth muscle cells.^7^ In addition, PGC‐1α is a co‐activator of PPAR‐γ. Therefore, we speculated that hypoxia can also down‐regulate the expression of PGC‐1α in skeletal muscles.^8^ Therefore, we will verify the pathological injuries of the palatopharyngeus muscle in OSA patients and explore the role of PGC‐1α in the changes in airway dilator structure and conversion of muscle fiber types in patients with OSA. Exploring the pathophysiological mechanism underlying OSA provides a new perspective for the early clinical treatment of OSA.

## 2 MATERIALS AND METHODS

Patients who were initially diagnosed with OSA and chronic tonsillitis and who underwent surgery in the Department of Otolaryngology Head and Neck Surgery at Sun Yat‐sen Memorial Hospital of Sun Yat‐sen University between August 2018 and August 2020 were included in this study. All patients underwent polysomnography (PSG). According to the diagnostic criteria in the diagnostic and treatment guidelines for OSA,^9^ apnea–hypopnea index (AHI) of ≥5 times each hour met the criteria for the diagnosis of OSA. The control group included patients with chronic tonsillitis without OSA. The study was approved by the hospital ethics committee, and all the enrolled patients signed an informed consent form.

Exclusion criteria: (1) patients with a history of oral, maxillofacial, and upper airway surgery; (2) patients with muscular diseases, such as systemic muscle weakness and muscular atrophy; (3) patients with systemic diseases involving muscles, such as vasculitis and connective tissue diseases; (4) patients who suffered cerebrovascular accidents or who had a history of radiotherapy or chemotherapy; and (5) patients with OSA who had been treated with CPAP, oral orthodontics or surgery in the past.

All OSA patients were grouped according to AHI and the lowest oxygen saturation (LSaO2).^9^ An AHI between 5 and 15 indicated mild disease, >15–30 indicated moderate disease, and >30 indicated severe disease. The LSaO2 < 0.9–0.85 indicated mild hyoxemia, <0.85–0.65 indicated moderate hyoxemia, and <0.65 indicated severe hyoxemia.

Tonsillectomies were performed in both groups, and two pieces of palatopharyngeal muscle tissue (approximately 4 × 4 × 3 mm in size) were collected. Avoid clamping or pulling the tissues and the tissue samples were stored in liquid nitrogen and glutaraldehyde.

### 2.1 Immunohistochemistry

Tissues were embedded in paraffin and cooled on a −20°C refrigerated table. Then, the tissues were sliced with a paraffin slicer to a thickness of 4 μm. The tissues were collected on glass slides and placed in a 60°C oven for baking. Then, dewaxing, hydration, antigen retrieval, membrane rupture, and other treatments were carried out. The cells were incubated with 10% goat serum to prevent nonspecific staining, and the following antibodies were added: rabbit anti‐human PGC‐1α antibody (Abcam), biotinylated goat anti‐rabbit IgG (Kangwei), and DAB color liquid (Beyotime). Finally, the tissue sections were stained with hematoxylin, dehydrated, made transparent, sealed with neutral resin, and naturally dried.

The sections were viewed and photographed using a light microscope connected to a computer. Each sample was consecutively photographed with a 200× magnification objective. Myofiber morphology, muscle fiber arrangement, PGC‐1α staining localization, and staining intensity were observed. The immunohistochemical results were semiquantitatively analyzed,^9^ and 5 fields were randomly selected for scoring and measuring the intensity of cell staining at 200× magnification. The scoring system was as following: brown scored as 3 points, tan scored as 2 points, and light yellow scored as 1 point. No coloring scored as 0 points.

### 2.2 Real‐time quantitative PCR

Tissues were cut and homogenized, and RNA was extracted. The concentration and purity of the RNA and synthesized cDNA were determined according to the instructions of the Beyort™III cDNA Synthesis Kit (BeyoTime) (The primer sequences were shown in Table 1). With cDNA as the template and GAPDH as internal reference, the relative mRNA expression levels of PGC‐1α and NRF‐1 in the palatopharyngeus muscle of the OSA and control groups were assessed according to the instructions of the qPCR kit (Beyotime).

**TABLE 1:** List of human primers used for quantitative PCR

Primer name	Sequence(5′–3′)
PGC‐1α	
F	CAAGCCAAACCAACAACTTTATCTCT
R	CACACTTAAGGTGCGTTCAATAGTC
NRF‐1	
F	GGGAGCTACAGTCACTATGGCG
R	ACAAGACGATCTGTCCCCCA
GAPDH	
F	CAGGGCTGCTTTTAACTCTG
R	TAGAGGCAGGGATGATGTTC

### 2.3 Western blotting

Tissues were collected and transferred to a EP tube. After grinding the tissues, lysis solution was added to extract the total proteins. Protein quantification was performed according to the instructions of the BCA protein concentration measurement kit (Zhongshan Jinqiao).

Thirty micrograms of each protein lysate was separated via a 10% SDS‐polyacrylamide gel (Beyotime) and transferred to a polyvinylidene difluoride membrane (Beyotime). After incubation in a blocking solution containing 5% skim milk for 1 h, the following primary antibodies were diluted and added to the membranes: PGC‐1α (1:1000, Abcam), NRF‐1 (1:1000; Kangweiji), and glyceraldehyde‐3‐phosphate dehydrogenase (GAPDH). The membranes were incubated at 4°C overnight. After being washed, the peroxidase‐conjugated secondary antibody (1:3000; goat anti‐rabbit IgG; Kangweiji) was diluted, added to the membranes, and incubated for 1 h at room temperature. The membranes were exposed to ECL solution, and the densities of the obtained bands were quantified by NIH ImageJ. GAPDH used as the internal control.

### 2.4 Electron microscopy

Tissues were immersed and fixed in glutaraldehyde (Alaaesar). Then, the tissues were cut into sections along the muscle fibers, fixed with 1% osmic acid (TED Pella, USA), dehydrated with ethanol and acetone, soaked with resin, embedded, and solidified with EP‐812 (TED Pella). The samples were positioned, and semi‐thin sections (70 μm thick) were generated with an ultrathin microslicer. The cells were stained with 2% uranium dioxide acetate (EMS, USA) and lead citrate (TED Pella). After film‐making, the samples were observed and images were captured under a transmission electron microscope.

### 2.5 Cell culture, hypoxic culture, and transient transfection

C2C12 myoblasts (CELLCOOK) were maintained in high‐glucose DMEM (Procell, Wuhan, China) containing 10% fetal bovine serum and 1% P/S in an incubator at 37°C in 5% CO_2_. When cells reached 80%–90% confluence, the medium was changed to differentiation medium (DMEM with 2% horse serum [ABSIN]).

When the C2C12 myoblasts had differentiated into muscular tubes, they were divided into 6 groups. Three groups were treated with intermittent exposure to hypoxia, and three groups (control) were treated with normoxia. The intermittent hypoxia conditions were as follows: the CO_2_ and O_2_ concentrations were controlled by N_2_, and the cells were exposed to hypoxia treatments for 8 h each day. Each hypoxic treatment included culture under hypoxic conditions for 35 min and culture under normoxic conditions for 25 min. The oxygen concentration was 5%, and the temperature, CO_2_ concentration, and saturated humidity were the same as those used for the control group.^10^

The cationic liposome method was used for transfection, and the procedure was performed according to the instructions of the LipofectamineTM 2000 kit (ReeboBio, Guangzhou). Cell qPCR and Western blotting followed the procedures described above (the primer sequences are shown in Table 2).

**TABLE 2:** List of mouse primers used for quantitative PCR

Primer name	Sequence(5′–3′)
PGC‐1α	
F	AGGAAATCCGAGCGGAGCTGA
R	GCAAGAAGGCGACACATCGAA
NRF‐1	
F	TCGGGCATTTATCCCAGAGATGCT
R	TACGAGATAAGCTATACTGTGTGT
GAPDH	
F	CCACATCGCTCAGACACCAT
R	CCAGGCGCCCAATACG

### 2.6 Immunofluorescence staining

Paraformaldehyde (Beyotime) was used to fix the slides, and 0.5% Triton X‐100 (Zhongshanqiao) was used for membrane permeabilization. The cells were incubated with 10% goat serum to prevent nonspecific staining, and the following antibodies were added: anti‐slow skeletal myosin heavy chain antibody and anti‐fast skeletal myosin heavy chain antibody. The sections were incubated with these antibodies at 4°C overnight. After washing, the goat anti‐rabbit IgG (KangWeishiji) secondary antibody was added and incubated at room temperature for 1 h. 4′,6‐diamidino‐2‐phenylindole (DAPI) (KangWeishiji) was added dropwise and incubated to stain the nuclei. The sections were sealed with anti‐fluorescence quenching agent, the sections were observed, and images were captured under a fluorescence microscope, and the densities of the images were quantified by NIH ImageJ.

### 2.7 Statistical analysis

SPSS 20.0 statistical software was used for the data analysis. All data were tested for normality and analysis of variance. Immunohistochemical results were semiquantitatively analyzed by an integral comprehensive method according to the cell staining intensity. The qPCR results are presented as Folds = $2^{-\Delta \Delta C_{t}}$ to present the relative expression level, and the Western blotting bands were quantified by ImageJ. The experimental data are expressed as the mean ± SD, and *p* < 0.05 was considered statistically significant.

## 3 RESULTS

### 3.1 Clinical features of patients and mRNA expression of PGC‐1α and NRF‐1

The study included 30 patients with OSA with a history of 1–20 years, including five patients with mild disease, four patients with moderate disease and 21 patients with severe disease, and 20 subjects were included the control group. In Table 3, we can see that the body mass index (BMI) of the OSA group was higher than that of the control group. The mRNA expression of PGC‐1α and NRF‐1 in the palatopharyngeus muscle of OSA patients was lower than that in the palatopharyngeus muscle of the controls. Then, the patients with OSA were classified according to the AHI and LSaO2. The BMI and PGC‐1α and NRF‐1 mRNA expression of the patients in the different AHI groups and LSaO2 groups were analyzed. Expression levels were compared among the groups. As shown in Tables 4 and 5, the BMI increased with increasing AHI and the severity of hypoxia, but there was no statistically significant difference in BMI among the different AHI groups (*p* = 0.540) and LSaO2 groups (*p* = 0.077). Our sample is small, especially for mild and moderate patients, which may affect the conclusion. With the aggravation of hypoxia, the mRNA expression of PGC‐1α and NRF‐1 decreased. A long‐term hypoxic environment down‐regulated PGC‐1α and NRF‐1 expression in the palatopharyngeus muscle of OSA patients, and the decrease in PGC‐1α expression was more pronounced in patients with severe OSA. We speculated that long‐term hypoxia led to a decrease in PGC‐1α expression and injury to the upper airway dilator muscle and exacerbated airway collapse and hypoxia.

**TABLE 3:** Clinical characteristics and the mRNA expression of PGC‐1α and NRF‐1

	OSA (n = 30)	Control (n = 20)	*p*
Age (year)	13–58 (37.67 ± 11.1)	20–63 (38.30 ± 11.48)	0.846
BMI (Kg/m2)	20.57–36.0 (27.89 ± 3.51)	17.31–28.41 (23.25 ± 3.17)	<0.001
Sex			
Male	28	16	
Female	2	4	0.155
PGC‐1α	0.447 ± 0.153	0.734 ± 0.183	<0.001
NRF‐1	0.476 ± 0.267	0.705 ± 0.337	0.010

Abbreviations: AHI, apnea–hypopnea index; BMI, body mass index; NRF‐1, nuclear respiratory factor‐1; PGC‐1α, peroxisome proliferator‐activated receptor‐γ coactivator‐1α.

**TABLE 4:** BMI and the mRNA expression of PGC‐1α and NRF‐1 in each AHI group

	Mild	Moderate	Severe	*p*
n
5	4	21	
Age (year)	32 ± 16.32	32.75 ± 4.35	40 ± 10.20	0.245
Sex				
Male	4	4	20	
Female	1	0	1	0.517
BMI (Kg/m^2^)	26.4 ± 2.70	27.5 ± 6.56	28.29 ± 2.85	0.540
PGC‐1α	0.65 ± 0.14	0.54 ± 0.06	0.38 ± 0.11	<0.001
NRF‐1	0.61 ± 0.21	0.65 ± 0.21	0.41 ± 0.27	0.124

Abbreviations: AHI: apnea–hypopnea index; BMI: body mass index; PGC‐1α: peroxisome proliferator‐activated receptor‐γ coactivator‐1α; NRF‐1: nuclear respiratory factor‐1.

**TABLE 5:** BMI and the mRNA expression of PGC‐1α and NRF‐1 in each LSaO2 group

	Mild	Moderate	Severe	*p*
n
4	13	13	
Age (year)	23.50 ± 7.59	40.54 ± 10.66	39.15 ± 9.56	0.031
Sex				
Male	3	13	12	
Female	1	0	1	0.314
BMI (Kg/m^2^)	25.18 ± 4.28	27.16 ± 2.83	29.45 ± 3.39	0.077
PGC‐1α	0.56 ± 0.09	0.49 ± 0.19	0.37 ± 0.09	0.021
NRF‐1	0.57 ± 0.22	0.67 ± 0.16	0.47 ± 0.13	0.054

Abbreviations: BMI, body mass index; LSaO2, lowest oxygen saturation; NRF‐1, nuclear respiratory factor‐1; PGC‐1α, peroxisome proliferator‐activated receptor‐γ coactivator‐1α.

### 3.2 Protein expression of PGC‐1α and NRF‐1 in patients with OSA

**FIGURE 1:**
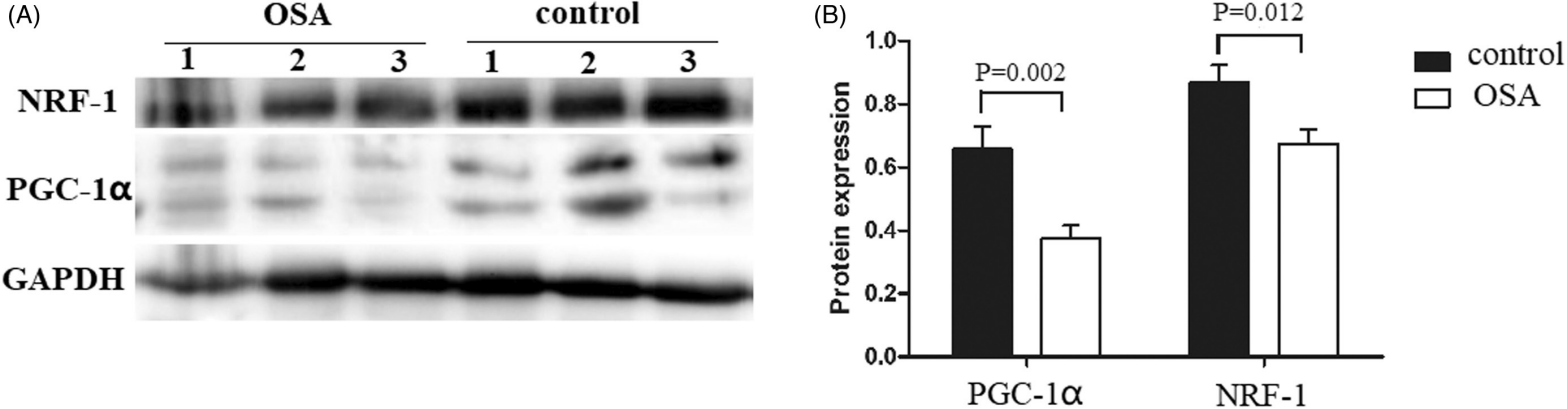
Western blotting result showed that the protein expression of PGC‐1α and NRF1 in OSA group was lower than that in control group

Western blotting was used to detect the protein expression of PGC‐1α and NRF‐1 in the OSA and control groups. The gray values of the bands were measured by ImageJ for statistical analysis. Compared with that in the control group, the protein expression of PGC‐1α (t = 3.555, *p* = 0.002) and NRF‐1 (t = 2.784, *p* = 0.012) in the OSA group was decreased (Figure 1).

### 3.3 Changes in muscle fiber structure in the palatopharyngeus muscle of patients with OSA

**FIGURE 2:**
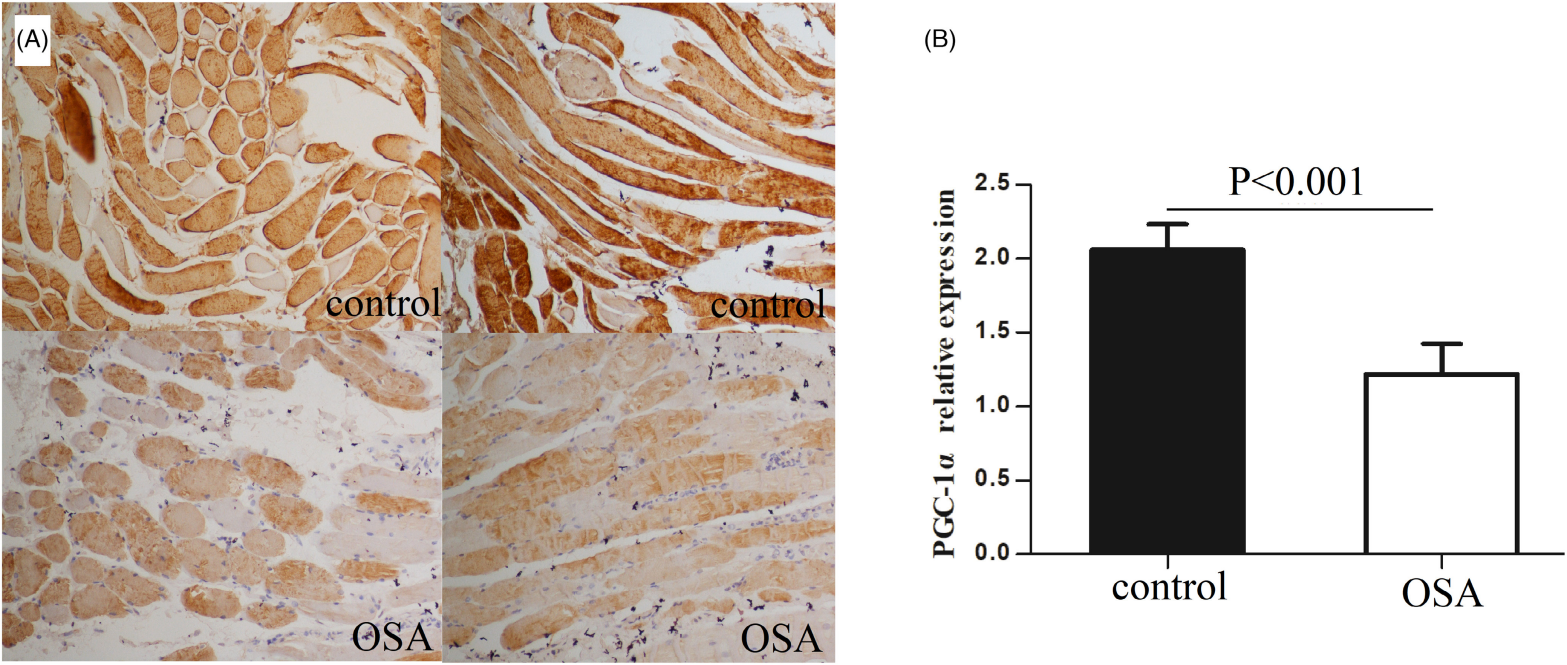
(A) Immunohistochemical staining was used to detect the changes in muscle fiber structure and PGC‐1α expression in upper airway dilator in OSA group and control group (magnification ×200). In the OSA group, the morphology of muscle fibers was changed, exhibited disordered muscle fiber structure, and increased connective tissue between cells, and the cells were lightly stained. In the control group, muscle fibers were arranged neatly, the cell morphology was regular and the staining was uniform. (B) The immunohistochemistry results were semiquantitatively analyzed, and rhe results showed that the expression of PGC‐1α in the OSA group was lower than that in the control group

To observe the structure of the palatopharyngeus muscle and the protein expression of PGC‐1α in muscle fibers, we used immunohistochemical staining to observe muscle structure under a microscope. The brown color indicated positive PGC‐1α protein staining. PGC‐1α expression was observed in both nucleus and cytoplasm. In the control group, cells were darkly stained, mainly brown and tan, and neatly arranged. In the OSA group, cells were lightly stained, mainly light yellow, and exhibited disordered muscle fiber structure and increased connective tissue between cells. The immunohistochemistry results were semiquantitatively analyzed according to the methods described above. The results indicated that the expression of PGC‐1α in the control group was higher than that in the OSA group (t = 8.321, *p* < 0.001) (Figure 2).

### 3.4 Electron microscopy to detect the muscle fiber structure of palatopharyngeus muscle of patients with OSA

**FIGURE 3:**
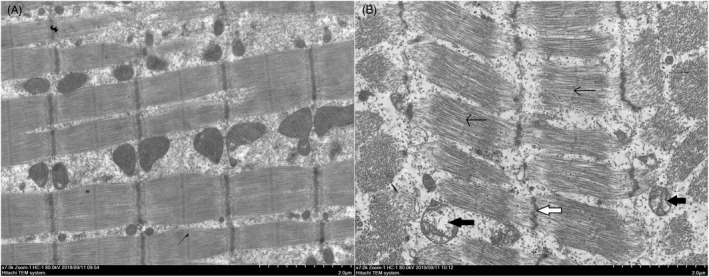
Muscle fiber structure of upper airway dilator in OSA group and control group was observed under electron microscope. (A) In the control group, muscle fibers were neatly arranged with clear structure and mitochondrial structure was normal. (B) In the OSA group, muscle fibers were disordered, with separation of muscle filaments, Z‐line distortion and partial disappearance (shown by white arrow), M‐line partial disappearance (shown by single arrow), reduced mitochondria and vacuole‐like degeneration in mitochondria (shown by black arrow)

Under an electron microscope, it was seen that the palatopharyngeus muscle of patients with OSA exhibited muscle atrophy, increased intermuscular tissue, disordered muscle fibers, broken and separated myofilaments, and unclear boundaries between bright and dark bands. The Z line and M line were distorted, and lost continuity and local interruptions were observed. The numbers of mitochondria were decreased, the morphology of mitochondria was changed, and the mitochondria were swollen and vacuolated (Figure 3).

### 3.5 The mRNA expression of PGC‐1α and NRF‐1 in C2C12 myoblasts cultured under intermittent hypoxic conditions

**FIGURE 4:**
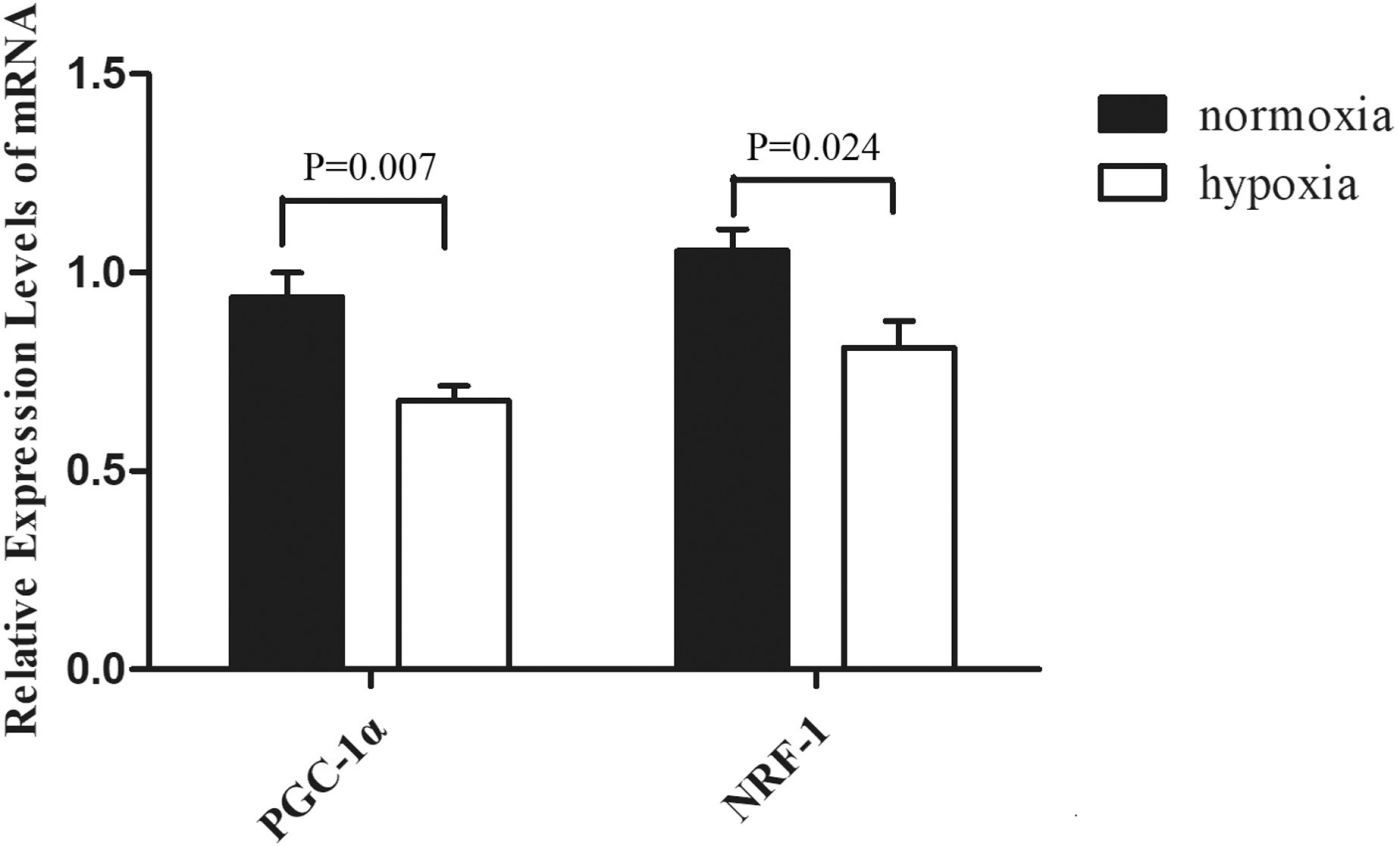
mRNA expressions of PGC‐1α and NRF‐1 in the hypoxia group were lower than those in the control group

The qPCR was used to detect the mRNA expression of PGC‐1α and NRF‐1 in cells in the hypoxia group and control group (normoxia). In the control group, the relative expression level of PGC‐1α was 0.938 ± 0.138; however, it was 0.677 ± 0.084 in the intermittent hypoxia group (t = 13.625, *p* = 0.007). The expression level of NRF‐1 was 1.055 ± 0.124 in the control group and 0.810 ± 0.152 in the intermittent hypoxia group (t = 2.791, *p* = 0.024). The difference was statistically significant (Figure 4).

### 3.6 The protein expression of PGC‐1α, NRF‐1, and type I and type II myofibril in C2C12 myoblasts cultured under intermittent hypoxic conditions

**FIGURE 5:**
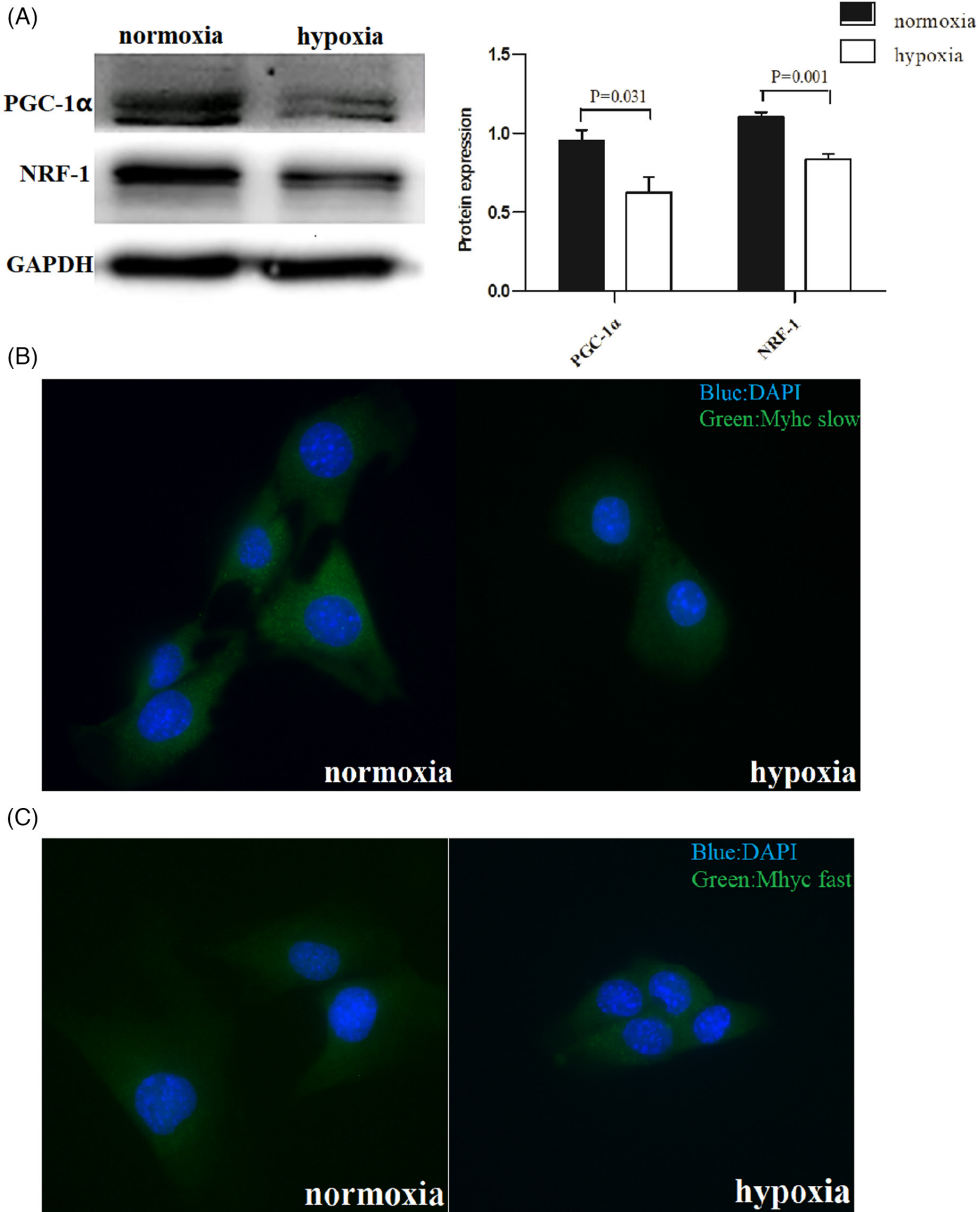
C2C12 myoblasts were cultured under normoxic and indirect hypoxic conditions, respectively. (A) The results of WB showed that the protein expressions of PGC‐1α and NRF‐1 in the hypoxia group were decreased compared with those in the control group. (B) Immunofluorescence staining results showed that the expression of type I myofibril in the hypoxia group was lower than that in the control group (blue: nuclear staining, green: type I myofibril staining). (C) Immunofluorescence staining results showed that the expression of type II myofibril in the hypoxia group was higher than that in the control group (blue: nuclear staining, green: type II myofibril staining)

The protein expression levels of PGC‐1α and NRF‐1 in the two groups were detected by Western blotting, and the expression of type I and type II myofibril was detected by immunofluorescence staining. Results showed that the protein expression of PGC‐1α and NRF‐1 in the intermittent hypoxia group was lower than that in the control group (Figure 5A). Compared with that in the control group (density: 0.231 ± 0.016), the expression of type I myofibril in the intermittent hypoxia group (density: 0.193 ± 0.006) was decreased(*p* = 0.001). Compare with the control group(density: 0.457 ± 0.036), the expression of type II myofibril inintermittent hypoxia group (density: 0.543 ± 0.035) was increased (*p* = 0.002)(Figure 5B,C).

### 3.7 The expression of NRF‐1, type I, and type II myofibril after PGC‐1α expression was knocked down

**FIGURE 6:**
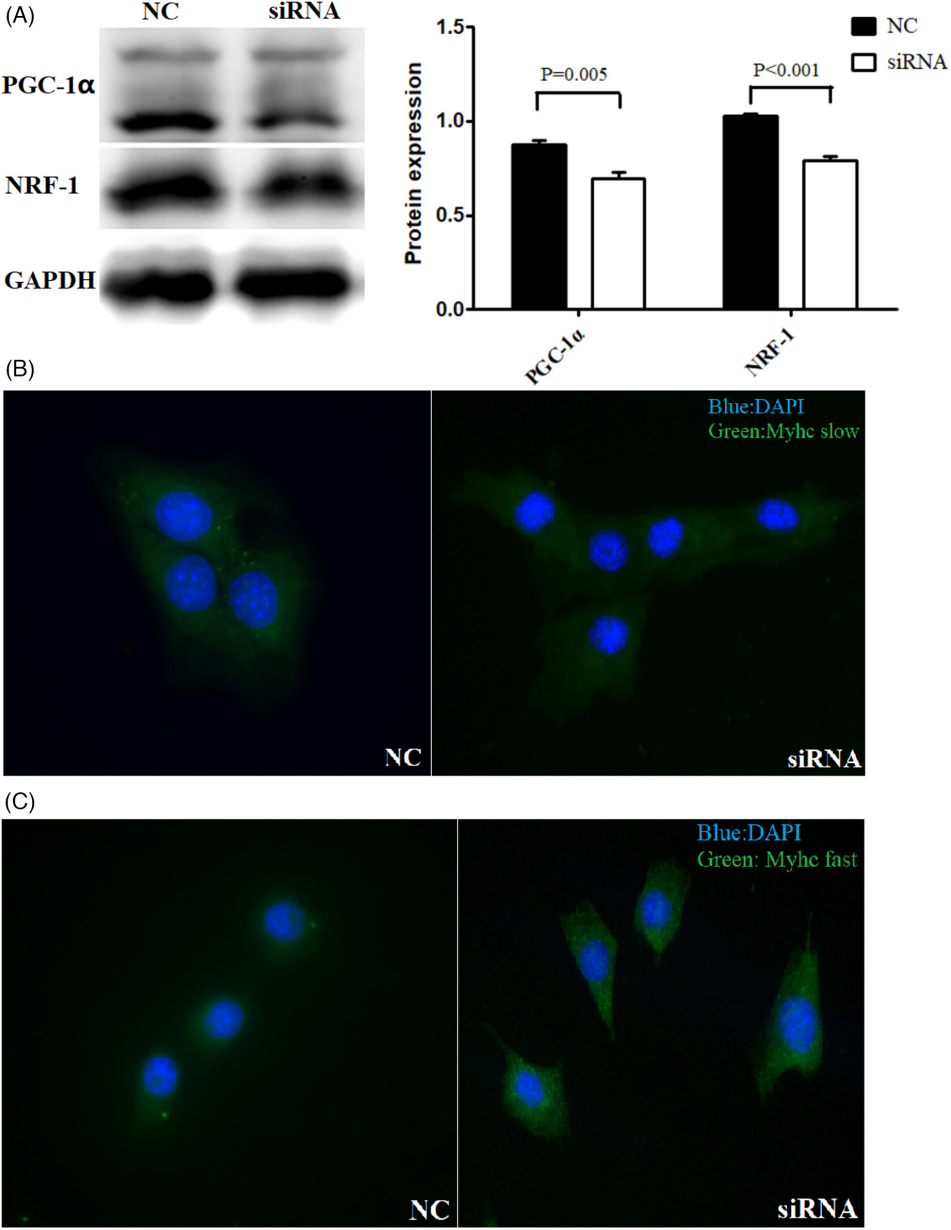
(A) PGC‐1α expression was knocked down by siRNA. The result of WB showed that the protein expression of NRF‐1 was down‐regulated (GPADH was used as internal reference). (B) Immunofluorescence staining showed that the expression of type I myofibril was decreased compared with control group (NC) (blue: nuclear staining, green: type I myofibril staining). (C) The expression of type II myofibril was increased compared with control group (NC) (blue: nuclear staining, green: type II myofibril staining)

PGC‐1α expression was knocked down by siRNA. The protein expression of NRF‐1 was detected by Western blotting, and type I, and II myofibril was detected by immunofluorescence staining. The protein expression of NRF‐1 was decreased after knocking down PGC‐1α expression (Figure 6A). Compared with the control group, knocking down PGC‐1α decreased the expression of type I myofibril (*p* = 0.002) while the expression of type II myofibril was increased (*p* = 0.001)(Figure 6B,C).

## 4 DISCUSSION

Previous studies have shown that in OSA, palatopharyngeus muscle exhibit pathological injuries, including muscle fibers atrophy and type I fibers reduction,^4^, ^5^ which lead to changes in function of the palatopharyngeus muscle, collapse of the upper airway and exacerbation of hypoxia. To explore the role of PGC‐1α in the structural changes and the conversion of muscle fiber type of palatopharyngeus muscle in OSA. We investigated the differences in palatopharyngeus muscle structure and expression of PGC‐1α in muscle fibers between OSA and control group. And investigated the mechanism of PGC‐1α mediating muscle fiber type conversion under repeated intermittent hypoxia. The result of our study showed that the palatopharyngeus muscle of patients with OSA became structural disordered, and the expression of PGC‐1α in muscle fibers was lower than that in the control group. With the severity of OSA disease, the mRNA expression of PGC‐1α was lower. In cell experiments, our results showed that PGC‐1α was involved in the conversion of palatopharyngeus muscle fiber types induced by intermittent hypoxia.

Chronic intermittent hypoxia down‐regulated the expression of PGC‐1α in skeletal muscle fibers. In this study, we found that the expression of PGC‐1α in the upper airway dilator muscle of OSA was lower than that of the control group. According to AHI, patients in the OSA group were divided into mild, moderate, and severe group, and the expression of PGC‐1α in palatopharyngeus muscle in the three groups were compared, respectively. It was found that the expression of PGC‐1α decreased gradually with the aggravation of OSA.

PGC‐1α is an effective transcription activator of nuclear receptors and other transcription factors. PGC‐1α is abundantly expressed in skeletal muscle, especially in type I muscle fibers.^11^ Stimuli, such as hypoxia, cold, and starvation, can affect the expression of PGC‐1α.^12^ Previous studies have shown that hypoxia stimulation can down‐regulate the expression of PPAR‐γ in smooth muscle,^7^ and PGC‐1α is its co‐activator. We speculated that hypoxia may also down‐regulate the expression of PGC‐1α. Our study verified that the expression of PGC‐1α was decreased in the palatopharyngeus muscle of patients with OSA by qPCR and Western blotting.

PGC‐1α can strongly induce mitochondrial biosynthesis. As a transcription factor co‐activator, PGC‐1α depends on transcription factors to function. NRF‐1 is a key factor in mitochondrial biosynthesis. NRF‐1 can participate in the regulation of mitochondrial respiratory chain complex synthesis and can affect the expression of mitochondrial DNA. Down‐regulation of the expression of NRF‐1 inevitably leads to a decrease in the number of mitochondria.^13^, ^14^, ^15^, ^16^ The regulation of mitochondria by PGC‐1α is mainly achieved by affecting the expression of NRF‐1. The results of this study also found that the expression of NRF‐1 is down‐regulated in palatopharyngeus muscle of patients with OSA, but the relationship between the down‐regulation of NRF‐1 and PGC‐1α expression and the conversion of palatopharyngeus muscle in patients with OSA remains to be further explored.

Studies have found that patients with OSA have disordered muscle structures, ruptured muscle filaments, motor nerve fiber edema, reduced mitochondrial numbers, and vacuolated mitochondria, which result in weakened muscle contractility.^17^ Especially after a long period of muscle contraction, the decrease in contractility was larger in OSA patients than in normal subjects. This finding indicated that the anti‐fatigue ability of the palatopharyngeal muscle is weakened in OSA patients.^4^ In addition, the palatopharyngeal muscle in patients with OSA also had muscle fibers of different sizes and increased connective tissue between muscle tissues. Connective tissue lacks the ability to contract, which results in palatopharyngeal muscle hypertrophy and reduced contractile function. The pathological changes in the palatopharyngeal muscle were positively correlated with the severity of OSA.^4^ Immunohistochemistry was used to observe the structural changes in the palatopharyngeus muscle fibers in the OSA and control groups. The results showed that the muscle cells in the control group were regular in shape and size, neatly arranged, tightly connected, and expressed abundant PGC‐1α protein levels. In the OSA group, the arrangement of muscle fibers was disordered, the connective tissue between cells was increased, and the expression of PGC‐1α was decreased. Electron microscopy also showed that compared with those in the control group, the palatopharyngeus muscle in the OSA group had muscle atrophy, disordered arrangement, and increased myofilament spacing. The Z lines and M lines are zigzagged, distorted, unclear, and even absent. The number of mitochondria decreased, the structure changed, and vacuolation degeneration occurred. By comparing the BMI in the OSA and control groups, we found that BMI in the OSA group was higher than that in the control group. The BMIs of the patients in the different AHI groups were analyzed. Results showed that the AHI increased with BMI. However, the difference in BMI among patients with mild, moderate, and severe OSA were not significant. Considering our small sample size, especially for mild and moderate patients, this may affect the conclusion. A larger number of patients is needed for further validation. The collapse of the palatopharyngeus muscle plays an important role in the progression of the disease. Under normal conditions, upper airway dilator tissue contains a specific proportion and arrangement of muscle fiber composition. The palatopharyngeus muscle of patients with OSA showed atrophy, disordered muscle fibers, and increased numbers of fibroblasts. Fibroblasts do not have contractile function, which results in reduced pharyngeal muscle contractile function and the inability to maintain an open upper airway.^18^, ^19^

Next, we cultured C2C12 myoblasts and exposed them to intermittent hypoxia to simulate the nocturnal hypoxia experienced by patients with OSA. Results showed that the levels of PGC‐1α and NRF‐1 in the C2C12 myoblasts cultured under intermittent hypoxic conditions were lower than those in the C2C12 myoblasts cultured under control conditions. In addition, immunofluorescence staining was used to detect the expression of type I and type II myofibril in the two groups. Results showed that the expression of type I myofibril in intermittent hypoxia group was also lower than that in the control group, and the expression of type II fibrin was increased and was synchronized with PGC‐1α expression. To elucidate whether the protein expression of NRF‐1, type I fibers, and type II fibers was regulated by PGC‐1α, we further used siRNA to knock down the expression of PGC‐1α. Results showed that with the decrease in PGC‐1α expression, the expression of NRF‐1 and type I myofibril decreased, and the expression of type II myofibril increased. Therefore, at the cellular level, we confirmed that PGC‐1α was involved in the muscle fiber type conversion caused by intermittent hypoxia, and NRF‐1 may be one of the important influencing factors.

However, Michael J and Dantas have showed that, excluding obesity‐related diseases, collagen type I was increased in the oropharyngeal muscles of OSA patients and was significantly associated with age. Increased collagen type I may lead to incomplete relaxation of the suprapharyngeal constrictor after expiration of the expiratory phase, which delay the contraction–relaxation response of the suprapharyngeal constrictor during the expiration‐inspiratory phase transition, thereby increasing pharyngeal collapsability. Therefore, those studies does not support the treatment of OSA by increasing upper airway collagen type I at the histological level.^20^, ^21^

The opening and closing of the upper airway is controlled by a complex interplay of pharyngeal muscles. The cause of increased pharyngeal structural collapse in OSA patients is unknown. Our study provides a preliminary idea into the mechanism of muscle fiber structural conversion, which still needs further exploration.

## 5 CONCLUSION

Pathological injuries were observed in the palatopharyngeus muscle of patients with OSA, including changes in the structure and arrangement of muscle fibers. The expression of PGC‐1α and NRF‐1 was down‐regulated, which became more obvious as the disease worsened. In cell experiments, intermittent hypoxia treatment decreased the expression of PGC‐1α, NRF‐1, and type I myofibril. When PGC‐1α expression was knocked down, the expression of NRF‐1 and type I myofibril was decreased. This result indicated that PGC‐1α was involved in the conversion of palatopharyngeus muscle fiber types caused by intermittent hypoxia.

## CONFLICT OF INTEREST

The authors declare that they have no competing interests.

## Data Availability

The article has no associated data.

## References

[1] Vasu TS , Grewal R , Doghramji K . Obstructive sleep apnea syndrome and perioperative complications: a systematic review of the literature. J Clin Sleep Med. 2012;8:199‐207. 22505868 10.5664/jcsm.1784 PMC3311420

[2] Zamarron C , Carcia Paz V , Riveiro A . Obstructive sleep apnea syndrome is a systemic disease. Current evidence. Eur J Intern Med. 2008;19:390‐398. 18848171 10.1016/j.ejim.2007.12.006

[3] Shi S , Xia Y , Chen S , et al. The relationship between structural/MHC changes in upper airway palatopharyngeal muscle morphology and obstructive sleep apnea/hypopnea syndrome. Eur Arch Otorhinolaryngol. 2014;271:109‐116. 23632864 10.1007/s00405-013-2361-z

[4] Dong J , Niu X , Chen X . Injury and apoptosis in the palatopharyngeal muscle in patients with obstructive sleep apnea‐hypopnea syndrome. Med Sci Monit. 2020;26:e919501. 32221272 10.12659/MSM.919501 PMC7139195

[5] Smirne S , Iannaccone L , Ferini SM , et al. Muscle fiber type and habitual snoring. Lancent. 1991;337(8741):597‐599. 10.1016/0140-6736(91)91651-a 1671953

[6] Lin J , Wu H , Tarr PT , et al. Transcriptional co‐activator PGC‐1α drives the formation of slow‐twitch muscle fiber. Nature. 2002;418:797‐801. 12181572 10.1038/nature00904

[7] Gong K , Xing D , Li P , et al. Hypoxia induces downregulation of PPAR‐γ in isolated pulmonary arterial smooth muscle cells and in rat lung via transforming growth factor‐β signaling. Am J Physiol Lung Cell Mol Physiol. 2011;301:L899‐L907. 21926264 10.1152/ajplung.00062.2011 PMC3233825

[8] Efferson CL , Winkelmann CT , Ware C , et al. Downregulation of notch pathway by a gamma‐secretase inhibitor attenuates AKT/mammalian target of rapamycin signaling and glucose uptake in an ERBB2 transgenic breast cancer model. Cancer Res. 2010;70:2476‐2484. 20197467 10.1158/0008-5472.CAN-09-3114

[9] Gottlieb DJ , Punjabi NM . Diagnosis and management of obstructive sleep apnea: a review. JAMA. 2020;323:1389‐1400. 32286648 10.1001/jama.2020.3514

[10] Scarpulla RC . Nuclear control of respiratory chain expression by nuclear respiratory factors and PGC‐1‐related co‐activator. Ann N Y Acad Sci. 2008;1147:321‐334. 19076454 10.1196/annals.1427.006 PMC2853241

[11] Benton CR , Nickerson JG , Lally J , et al. Modest PGC‐1α overexpression in muscle in vivo is sufficient to increase insulin sensitivity and palmitate oxidation in subsarcolemmal, not intermyofibrillar, mitochondria. J Biol Chem. 2008;283:4228‐4240. 18079123 10.1074/jbc.M704332200

[12] Puigserver P , Wu Z , Park CW , Graves R , Wright M , Spiegelman BM . A cold‐inducible co‐activator of nuclear receptors linked to adaptive thermogenesis. Cell. 1998;92:829‐839. 9529258 10.1016/s0092-8674(00)81410-5

[13] Winklhofer KF . Parkin and mitochondrial quality control: toward assembling the puzzle. Trends Cell Biol. 2014;24(6):332‐341. 24485851 10.1016/j.tcb.2014.01.001

[14] Iguchi M , Kujuro Y , Okatsu K , et al. Parkin‐catalyzed ubiquitin‐ester transfer is triggered by PINK1‐dependent phosphorylation. J Biol Chem. 2013;288(30):22019‐22032. 23754282 10.1074/jbc.M113.467530 PMC3724655

[15] Kane LA . PINK1 phosphorylates ubiquitin to activate parkin E3 ubiquitin ligase activity. J Cell Biol. 2014;205(2):143‐153. 24751536 10.1083/jcb.201402104 PMC4003245

[16] Noriyuki M , Keiji T . The PARK2/parkin receptor on damaged mitochondria revisited—uncovering the role of phosphorylated ubiquitin chains. Autophagy. 2015;11(9):1700‐1701. 26213095 10.1080/15548627.2015.1071760 PMC4590609

[17] McGuire M , MacDermott M , Bradford A . Effects of chronic intermittent asphyxia on rat diaphragm and limb muscle contractility. Chest. 2003;123:875‐881. 12628891 10.1378/chest.123.3.875

[18] Boyd JH , Petrof BJ , Hamid Q , Fraser R , Kimoff RJ . Upper airway muscle inflammation and denervation changes in obstructive sleep apnea. Am J Respir Crit Care Med. 2004;170(5):541‐546. 15151922 10.1164/rccm.200308-1100OC

[19] Saboisky JP , Stashuk DW , Hamilton‐Wright A , et al. Neurogenic changes in the upper airway of patients with obstructive sleep apnea. Am J Respir Crit Care Med. 2012;185:322‐329. 22016445 10.1164/rccm.201106-1058OC PMC3297112

[20] Dantas DA , Mauad T , Silva LF , et al. The extracellular matrix of the lateral pharyngeal wall in obstructive sleep apnea. Sleep. 2012;35:483‐490. 22467986 10.5665/sleep.1730 PMC3296790

[21] Brennick MJ . Examination of the pharyngeal muscle extracellular matrix offers new clues to pathogenesis in obstructive sleep apnea syndrome. Sleep. 2012;35:449‐450. 22467979 10.5665/sleep.1716 PMC3296783

